# Impairment of exocytotic transmitter release by decynium-22 through an inhibition of ion channels

**DOI:** 10.3389/fphar.2023.1276100

**Published:** 2023-10-10

**Authors:** Gabriele Haar, Katsiaryna Hrachova, Tanja Wagner, Stefan Boehm, Klaus Schicker

**Affiliations:** Division of Neurophysiology and Neuropharmacology, Centre of Physiology and Pharmacology, Medical University of Vienna, Vienna, Austria

**Keywords:** rat superior cervical ganglion, voltage activated Ca^2+^ channel, action potential, exocytotic transmitter release, 1-methyl-4-phenylpyridinium, decynium-22

## Abstract

**Introduction:** In addition to members of the family of Na^+^/Cl^−^ dependent monoamine transporters, organic cation transporters (OCTs), in particular OCT3, as well as the plasma membrane monoamine transporter (PMAT) may contribute to neuronal reuptake of according neurotransmitters. As opposed to the numerous blockers of monoamine transporters, only a very limited number of specific blockers of OCT3 and PMAT are available. In fact, decynium-22 is the only blocking agent with micromolar affinities for both transport proteins, and this molecule is frequently used to establish roles of OCT3 and/or PMAT as targets for antidepressant drugs and psychostimulants, respectively.

**Methods/Results:** To test for a function of these transporters in the sympathetic nervous system, uptake and release of [3H]1-methyl-4-phenylpyridinium (MPP^+^) was investigated in primary cultures of rat superior cervical ganglia. Uptake was reduced by cocaine or desipramine, blockers of the noradrenaline transporter, by about 70% and by corticosterone or β-estradiol, blockers of OCT3, by about 30%; decynium-22 achieved complete inhibition of uptake with half maximal effects at 3 μM. Depolarization dependent release was enhanced by corticosterone or β-estradiol, but reduced by decynium-22. As the latter effect is unlikely to be related to actions at OCT3 and/or PMAT, electrophysiological recordings were performed to reveal that decynium-22 inhibits action potential firing and currents through voltage activated calcium channels in superior cervical ganglion neurons.

**Discussion:** These results demonstrate that decynium-22 can impair exocytotic neurotransmitter release by interfering with several types of ion channels. Such transporter-independent effects of decynium-22 that my interfere with basic neuronal functions need to be considered when interpreting results obtained with decynium-22 as prototypic inhibitor of transmitter reuptake via OCT3 and/or PMAT.

## 1 Introduction

Chemical neurotransmission is terminated by two fundamentally distinct mechanisms that remove active neurotransmitters from the extracellular space: enzymatic extracellular degradation and transmembrane cellular uptake, respectively. Drugs that target either of these two diverging mechanisms exert pronounced therapeutic and/or toxic actions in the nervous system and can be employed to treat, for instance, Alzheimer’s disease and major depression, respectively (Dale et al., 2015; Giacobini et al., 2022).

Extracellular catecholamines were shown to be taken up into neurons and non-neural cells such as cardiomyocytes by two separate mechanisms that display high and low affinities for the substrates, respectively; these two mechanisms have been termed uptake_1_ and uptake_2_ (Iversen, 1965; Eisenhofer, 2001). The transmembrane proteins mediating uptake_1_ and _2_ are members of the family of Na^+^/Cl^−^ dependent monoamine neurotransmitter transporters, on the one hand, and organic cation transporters (OCTs) as well as the plasma membrane monoamine transporter (PMAT), on the other (Koepsell, 2021). Neither is the expression of the noradrenaline (norepinephrine transporter, NET) and dopamine (DAT) transporters confined to neurons, nor is that of OCTs and PMAT found in non-neuronal cells exclusively (Eisenhofer, 2001; Koepsell, 2021). Thus, uptake_1_ and uptake_2_ proteins can both operate even within one single cell.

While numerous inhibitors selectively blocking one of the monoamine transporters are available (Kristensen et al., 2011), the number of OCT and PMAT blockers is rather limited (Koepsell, 2021). In fact, the only agent that blocks OCTs and PMAT with affinities in the sub-to low micromolar range is decynium-22 (D22) (Bönisch, 2021; Koepsell, 2021). Nevertheless, several substrates and blockers of monoamine transporters that are used as psychoactive drugs of abuse (e.g., amphetamine, cocaine) or antidepressants (e.g., amitriptyline, fluoxetine, imipramine, sertraline) interact with at least one of the three OCTs and/or PMAT at concentrations of about 10 µM or even below (Bönisch, 2021; Koepsell, 2021). Accordingly, it has been hypothesized that OCTs and/or PMAT might contribute to actions of amphetamine-like drugs and antidepressants. To clarify these issues, D22 has been tested for antidepressant actions in mice (Horton et al., 2013; Bowman et al., 2020) and rats (Marcinkiewcz and Devine, 2015) and has been employed to assess a role of OCT3 in the psychostimulant actions of amphetamine (Mayer et al., 2018).

Primary cultures of dissociated rat superior cervical ganglia (SCG) are frequently used as model system to investigate various aspects of neurotransmitter release. In this respect, this preparation is better suited than, for instance, tissue slices containing axon terminals of sympathetic ganglia as a contribution of target cells such as cardiomyocytes to the results obtained can be excluded (Boehm and Huck, 1997). Moreover, the dissociated neurons in cell culture are easily accessible for electrophysiological experiments in order to complement data concerning transmitter release with measurements of membrane excitability and ion channel currents. SCG neurons were reported to express OCT3 (but not OCT1 and 2), and this transporter was found to mediate efflux of MPP^+^ from the neurons when liberated from storage vesicles by, for instance, reserpine (Kristufek et al., 2002). In addition, D22 has been described to reduce amphetamine induced release of MPP^+^ from SCG neurons in a concentration dependent manner, and this was taken as evidence for the contribution of OCT3 to the psychostimulant effects of this amine (Mayer et al., 2018).

A question that has remained unresolved concerns a potential role of OCTs when transmitter release from SCG neurons is triggered by action potentials rather than vesicle depletion or amphetamine exposure. To clarify this, the present experiments investigated effects of D22 on (i) uptake of [^3^H]MPP^+^ into SCG cultures and (ii) electrically evoked release of previously incorporated [^3^H]MPP^+^ from such cultures. Respective results obtained with D22 were compared with those of corticosterone and ß-estradiol. In addition, effects of D22 on action potentials and voltage activated Ca^2+^ currents were analyzed in patch-clamp experiments. The results indicate that unspecific actions of D22 on ion channels may occlude effects elicited by an inhibition of transporters.

## 2 Material and methods

### 2.1 Preparation of SCG cultures

Primary cultures of dissociated rat SCG were prepared as described previously (Mosshammer et al., 2022). Sprague-Dawley rats were kept under appropriate conditions and sacrificed by decapitation at postnatal days 5–8 in full accordance with the rules of the Austrian animal protection and animal experiment law: (https://www.ris.bka.gv.at/Dokumente/BgblAuth/BGBLA_2004_I_118/BGBLA_2004_I_118.html) (https://www.ris.bka.gv.at/Dokumente/BgblAuth/BGBLA_2012_I_114/BGBLA_2012_I_114.html). According to regulations of the University and Austrian law, this procedure does not require an ethical approval. Immediately after decapitation, ganglia were removed, cut into two pieces, and incubated in a mixture of collagenase (1.5 mg·ml^−1^, Sigma-Aldrich, Vienna, Austria) and dispase (3.0 mg·ml^−1^, Roche, Vienna, Austria) for 30 min at 37°C, followed by an incubation in trypsin (0.25%, Worthington, Lakewood NJ, United States) for 15 min at 37°C. Ganglia were washed twice with calcium free Tyrode solution [in mM: NaCl (150), KCl (4), MgCl2 (2), glucose (10), HEPES (10), pH 7.4 adjusted with NaOH] followed by mechanical dissociation via trituration in Dulbecco’s modified Eagle’s Medium (DMEM, Sigma-Aldrich, Vienna, Austria) containing 10 mg·l^−1^ insulin (Sigma-Aldrich, Vienna, Austria), 50 μg·l^−1^ nerve growth factor (R&D Systems Minneapolis MN, United States), 25.000 IU·l^−1^ penicillin (Sigma-Aldrich, Vienna, Austria) and 25 mg·l^−1^ streptomycin (Sigma-Aldrich, Vienna, Austria). Depending on the type of experiment to be carried out, cells were plated in the following ways: (i) for electrophysiological experiments, 50,000 cells were seeded into 5 mm diameter glass rings placed in rat tail collagen coated (Trevigen, Minneapolis MN, United States) 35 mm tissue culture dishes (ThermoFisher scientific, Waltham MA, United States); (ii) for release experiments, 45,000 cells were seeded onto 5 mm rat tail collagen coated plastic discs (diameter 5 mm); (III) for radioactive uptake assays, 100,000 cells per well were seeded into rat tail collagen coated 48 well plates (Sarstedt, Nümbrecht, Germany). In all cases, 5% heat inactivated fetal bovine serum (Biowest, Nuaillé, France) was added after 2 h and cultures were stored for 4–6 days in a humidified 5% CO_2_ atmosphere at 37°C. Medium was exchanged on the 1st day after dissociation. Release cultures were changed to serum free medium on the day before the experiment. (iv) To assess potential uptake into non-neural cells present in routine SCG cultures, neurons were removed by first plating the cell suspension into uncoated 10 cm dishes. After 3 h, the supernatant containing the neurons was removed and fresh medium lacking NGF was added. After 5 days *in vitro*, cells were detached by trypsinization (Sigma Aldrich, Vienna, Austria), centrifuged at 80 g for 5 min, and resuspended in NGF free medium. Then, 100,000 cells per well were seeded into collagen coated 48 well plates and used for uptake experiments 5 days later.

### 2.2 Determination of uptake of [^3^H]1-methyl-4-phenylpyridinium (MPP^+^)

Prior to the experiment, cultures were washed with 800 µL buffer consisting of (in mM): NaCl (140), glucose (20), HEPES (10), CaCl_2_ (2.5), MgCl_2_(2), KOH (3), NaOH (2). Cells were then preincubated in buffer containing either β-estradiol (E2), corticosterone, decynium-22 (D22), cocaine or desipramine for 10 min. To compensate for effects of solvent, all solutions contained 0.25% DMSO (Sigma-Aldrich, Vienna, Austria). Thereafter, preincubation solutions were removed and uptake was initiated by addition of 100 µl of buffer containing 50 nM [3H]MPP^+^ (specific activity: 80 Ci·mmol^−1^, American Radiolabeled Chemicals, St. Louis, MO, United States) plus 10 µM non-tritiated MPP^+^ (Sigma-Aldrich, Vienna, Austria). After 5 min, uptake was terminated by the addition of 800 µl of ice cold buffer to each well and putting the plates on ice. Cells were washed with 800 µl of ice cold buffer once and lysed by addition of 1% sodium dodecyl sulfate. Radioactivity contained in these samples was assessed by liquid scintillation counting (Packard Tri-Carb 2800 TR, PerkinElmer, Brunn am Gebirge, Austria).

### 2.3 Determination of release of [^3^H]MPP^+^

Loading of SCG cultures with radiotracer and subsequent superfusion were performed as described before (Boehm, 1999). Culture discs were incubated in cell culture medium containing 27 nM [^3^H] MPP^+^ (specific activity: 80 Ci·mmol^−1^, American Radiolabeled Chemicals, St. Louis MO, United States) and 1 mM ascorbic acid for 60 min at 37°C. Thereafter, these discs were transferred into small chambers equipped with 2 platinum electrodes and continuously superfused with buffer consisting of (in mM): fumaric acid (0.5), Na-pyruvate (5), glucose (20), HEPES (10), ascorbic acid (0.57), NaCl (120), KCl (3), MgCl_2_ (2), CaCl_2_ (2), pH adjusted to 7.4 with NaOH, at a rate of approximately 1 ml·min^−1^ at 25°C. Collection of 4 min fractions of superfusate was started after a 60 min washout period during which excess radioactivity had been removed.

Depolarization-dependent tritium overflow was triggered either by electrical fields or by 50 mM KCl (NaCl was reduced accordingly to maintain isotonicity), both being applied for periods of 60 s. Stimulation by monophasic rectangular electrical pulses (0.5 ms, 60 mA, 50 V·cm^−1^) at 10 Hz or exposure to 50 mM K^+^ were performed at minutes 76 (S_1_) and 96 (S_2_), respectively. From minute 88 onward, the superfusion buffer contained solvent, corticosterone, β-estradiol, or D22. The radioactivity remaining in the cultures after the completion of experiments was extracted by immersion of the discs in 2% perchloric acid followed by sonication. Radioactivity in extracts and collected fractions was determined by liquid scintillation counting as above.

The rate of spontaneous (unstimulated) [^3^H] efflux was obtained by expressing the radioactivity retrieved during a collection period as a fraction of the total radioactivity in the cultures at the beginning of this period (fractional [^3^H] outflow). Stimulation-evoked tritium overflow was calculated as the difference between the total tritium outflow during and after stimulation and the estimated basal outflow, which was assumed to follow a linear time course throughout experiments. Therefore, basal outflow during periods of stimulation was assumed to equate to the arithmetic mean of the samples preceding and those following stimulation, respectively. Differences between total and estimated basal outflow during periods of stimulation were expressed as percentages of total radioactivity in the cultures at the onset of stimulation (% of total radioactivity; S%). The amount of radioactivity in the cultures at the beginning of each collection period is calculated by summing up the radioactivity remaining in the cells at the end of experiments and that retrieved during the respective and all subsequent collection periods.

As the amount of depolarization induced tritium overflow may vary considerably between different cultures (Scholze et al., 2002), the effects of transport inhibitors on depolarization-dependent release were evaluated by determining changes in the ratio of tritium overflow evoked during the two periods of electrical or K^+^ stimulation (S_2_/S_1_). Changes in spontaneous tritium outflow were analyzed in an analogous manner by calculating the ratio between the last fraction and the fraction preceding S_1_ (L_L_/L_1_). In Figure 3, values obtained in presence of varying concentrations of D22 were normalized to the values obtained in presence of solvent in the very same experiment.

### 2.4 Electrophysiology

All recordings were performed at room temperature (20°C–24°C) on the somata of single SCG neurons using the perforated patch-clamp technique (Hamill et al., 1981) to avoid dilution of intracellular molecules. Patch pipettes were pulled using a Flaming/Brown Puller (P97, Sutter Instruments, Novato CA, United States) from borosilicate glass capillaries (Science Products, Frankfurt/Main, Germany), front filled with pipette solution and backfilled with the same solution containing 200 μg·ml^−1^ amphothericin B (Pan Reac AppliChem, Darmstadt, Germany). This routinely resulted in series resistances in the range of 10–20 MΩ 30 min after the establishment of a giga seal which were compensated for by 60% in voltage clamp experiments. Experiments were performed using a MultiClamp 700B patch clamp amplifier (Molecular Devices, San Jose CA, United States). Current traces were filtered with a 6 kHz 8 pole Bessel filter and digitized at 50 kHz using an Axon Digidata1440 interface (Molecular Devices, San Jose CA, United States). Voltage traces were filtered using a 10 kHz Bessel filter and were digitized at 20 kHz. During recordings, cells were continuously superfused using a DAD-12 drug application device (ALA Scientific, NY), which allows a complete exchange of solutions within less than 100 ms (Boehm, 1999).

Calcium currents were measured by voltage clamping SCG neurons to −80 mV using a bath solution consisting of (in mM): NaCl (120); TEA (20), CaCl_2_ (2.5), MgCl_2_ (2), HEPES (10); glucose (10); MgCl_2_ (2); tetrodotoxin (0.0005), pH 7.4 adjusted with CsOH and a pipette solution containing (in mM): CsCl (130), TEACl (20), CaCl_2_ (0.24), EGTA (5), glucose (10), HEPES (10), pH 7.2 adjusted with CsOH. Cells were depolarized to +10 mV for 30 ms once every 15 s. The charge transfer during depolarization was measured as the area under the curve of the current trace. As amplitudes of calcium currents in different SCG neurons display huge variations, charge transfer in presence of D22 was expressed as percentage of that in presence of solvent in the very same cell (% of control).

To determine membrane potential, neurons were current clamped using a pipette solution consisting of (in mM): K_2_SO_4_ (75); KCl (55); MgCl_2_ (8); HEPES (10); adjusted to pH 7.4 with KOH and a bath solution consisting of (in mM): NaCl (140); glucose (20); HEPES (10); CaCl_2_ (2.5); MgCl_2_ (2); KOH (3); NaOH (2), adjusted to pH 7.4 with NaOH. The calculated liquid junction potential of 8 mV was corrected for at the beginning of the experiment. To asses excitability in SCG neurons, 2 s long current steps (50–300 pA for 2 s in 50 pA increments once every 10 s) were applied and the sum of action potentials elicited by these 6 current steps was counted.

### 2.5 Statistics

Unless otherwise specified, all values represent means ± standard error of the mean. Non-linear fit results are reported as fit values plus respective 95% confidence intervals. N values represent (i) single wells in uptake assays, (ii) single culture discs in release assays, and (iii) single cells in electrophysiological experiments, respectively. Each experiment was conducted using at least three independent preparations of primary cultures. Statistical significance between two groups was tested by Mann-Whitney U-tests and between multiple groups by Kruskall-Wallis tests followed by Dunn’s multiple comparison using Graphpad Prism 5 (GraphPad Software, Bosten MA, United States). *p* values < 0.05 were taken as indication of statistical significance. Figures were prepared using Lualatex and the pgfplots package (https://github.com/pgf-tikz/pgfplots).

### 2.6 Materials

Bulk chemicals were purchased from Sigma-Aldrich (Vienna, Austria). [^3^H] 1-methyl-4-phenylpyridinium (MPP^+^) was purchased from American Radiolabeled Chemicals (St. Louis, MO, United States) and non-tritiated MPP^+^ from Sigma-Aldrich (Vienna, Austria). Corticosterone, β-estradiol, and D22 were obtained from Sigma-Aldrich (Vienna, Austria), THP (Vienna, Austria) and Synthon Chemicals (Bitterfeld-Wolfen, Germany) respectively. Tetrodotoxin (TTX) was purchased from Latoxan (Valence, France).

## 3 Results

### 3.1 Effects of D22 on uptake of MPP^+^ into SCG neurons

**FIGURE 1:**
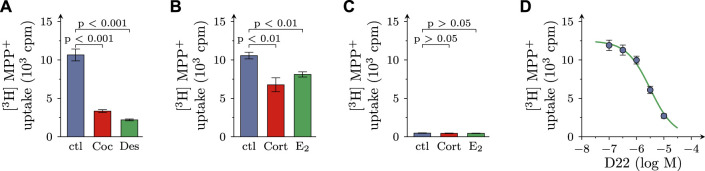
Effects of D22 on [^3^H]MPP^+^ uptake into SCG cultures: comparison with other uptake inhibitors. SCG cultures were exposed to 0.05 µM [^3^H]MPP^+^ plus 10 µM MPP^+^ for 5 min in presence of either solvent (control; ctl) or cocaine (Coc, 100 µM), desipramine (Des, 1 µM), corticosterone (Cort; 100 µM), β-estradiol (E2; 100 µM), and D22 (0.01–10 µM), respectively. **(A,B)** Radioactivity incorporated into cultures in presence of **(A)** solvent, cocaine, or desipramine (*n* = 18) and **(B)** solvent, corticosterone, or β-estradiol (*n* = 21), respectively. **(C)** Radioactivity incorporated into cultures lacking neurons in presence of solvent, corticosterone, and β-estradiol, respectively (*n* = 15). **(D)** Radioactivity incorporated into cultures in presence of the indicated concentrations of the D22 (*n* = 18). Significance of differences was evaluated by Kruskal-Wallis tests followed by Dunn’s multiple comparisons, and respective *p*-values are shown.

After incubation of SCG cultures in 0.05 µM [^3^H]MPP^+^ plus 10 µM MPP^+^ for 5 min, 11.75 ± 0.41 × 10^3^ cpm of radioactivity were retrieved within the cells (*n* = 57), which equates to 4.09% ± 0.13% of the amount of tritium the cultures had been exposed to. In the presence of either 100 µM cocaine or 1 µM desipramine, both blockers of NET, the incorporation of radioactivity was reduced by approximately 70%. In the presence of either 100 µM corticosterone or 100 µM β-estradiol, both blockers of OCT2 and 3 (Koepsell, 2021), the inhibition of uptake amounted to about 30% (Figures 1A, B).

For comparison, in cultures dissociated from SCG, but devoid of neurons, 0.48 ± 0.03 × 10^3^ cpm of radioactivity were incorporated in the cells (*n* = 15; *p* < 0.001 vs. cultures containing neurons), and this corresponded to 0.17% ± 0.01% of the amount of tritium the cultures had been incubated in. In neuron-free cultures, 100 µM corticosterone as well as 100 µM β-estradiol failed to cause any change in uptake (Figure 1C). Together, these results indicate that in primary SCG cultures MPP^+^ is incorporated by >95% into neurons, and not non-neural cells, and this neuronal uptake involves the NET as well as OCT3 (see also Kristufek et al., 2002).

In cultures containing neurons as well as non-neural cells, D22 reduced uptake in a concentration-dependent manner with a calculated value of half maximal inhibition at 3.2 (1.5–6.9) µM (*R* ^2^ = 0.7) and with 79.3% + 1.6% inhibition at 10 µM (Figure 1D).

### 3.2 Effects of D22 on spontaneous and action potential dependent release of MPP^+^ from SCG neurons

**FIGURE 2:**
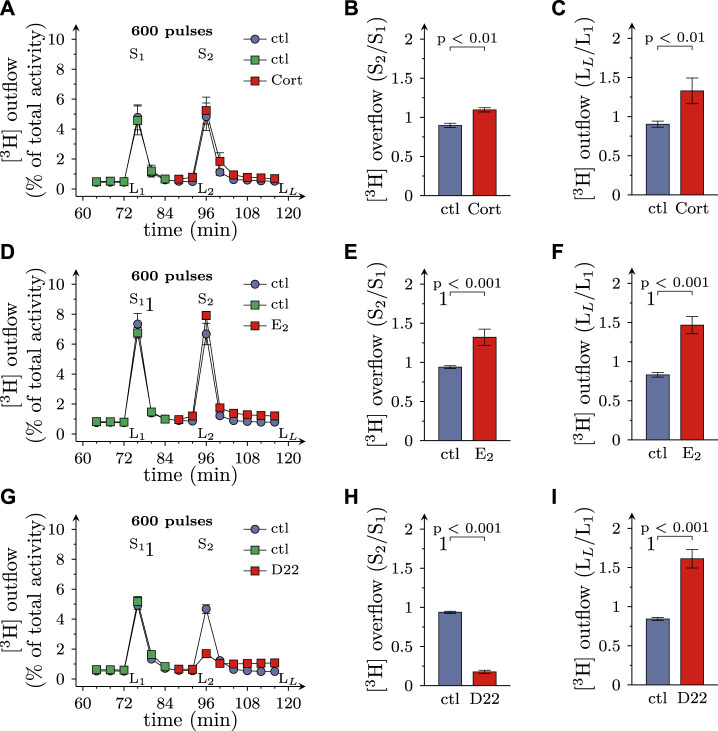
Effects of D22 on spontaneous and electrically evoked release of [^3^H]MPP^+^ from SCG neurons: comparison with corticosterone and β-estradiol. SCG cultures were labeled with [^3^H]MPP^+^ and superfused; subsequent to a 60 min washout period, 4 min fractions of superfusate were collected. Electrical field stimulation (600 pulses at 10 Hz) was applied in minutes 76 (S_1_) and 96 (S_2_), respectively. Corticosterone (Cort; 100 µM), β-estradiol (E2; 100 µM), D22 (3 µM), or solvent (control; ctl) were present from minute 84 onward as indicated. **(A,D,G)** show the time-course of [^3^H] outflow as determined in representative experiments (*n* = 3 each). **(B,E,H)** depict S_2_/S_1_ values of electrically evoked [^3^H] overflow in presence of solvent or uptake inhibitors as indicated (*n* = 12 for corticosterone and β-estradiol; *n* = 9 for D22). **(C,F,I)** depict L_L_/L_1_ values of spontaneous [^3^H] outflow (*n* = 21 for corticosterone; *n* = 12 for β-estradiol; *n* = 18 for D22; the data for corticosterone and D22 contain values of spontaneous release derived from experiments shown in Figure 6). Significance of differences was evaluated by Mann-Whitney U-tests, and respective *p* values are shown.

Excitation of SCG cultures by electrical field stimulation leads to action potential dependent vesicle exocytosis from axon terminals (Boehm, 1999), and blockage of monoamine transporters by, for instance, cocaine leads to an increase in electrically evoked transmitter overflow (Boehm et al., 1995). To assess a role of OCT3 in physiological transmitter release, cultures were exposed to 100 µM corticosterone and 100 µM β-estradiol, respectively. Both agents caused an augmentation of spontaneous as well as electrically evoked MPP^+^ efflux (Figures 2A–F). When 3 µM D22 was used instead, spontaneous release of MPP^+^ was elevated as before, but electrically evoked release became largely diminished (Figures 2G–I). Thus, the three blockers of OCT exerted diverging actions on action potential dependent transmitter release, with the steroids causing an enhancement and the decynium compound causing a reduction.

**FIGURE 3:**
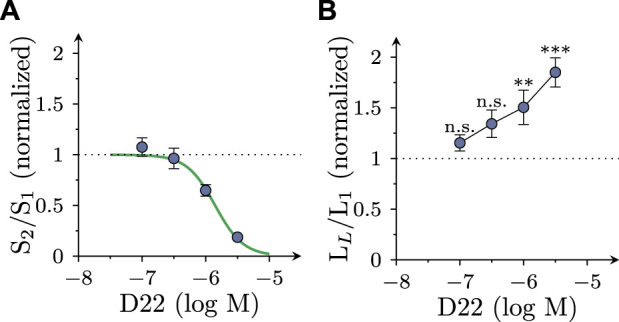
Concentration dependence of the effects of D22 on spontaneous and electrically evoked release of [^3^H]MPP^+^ from SCG neurons. SCG cultures were labeled with [^3^H]MPP^+^ and superfused; subsequent to a 60 min washout period, 4 min fractions of superfusate were collected. Electrical field stimulation (600 pulses at 10 Hz) was applied in minutes 76 (S_1_) and 96 (S_2_), respectively. D22 or solvent were present from minute 84 onward. **(A)** Depicts normalized S_2_/S_1_ values of electrically evoked [^3^H] overflow in presence of the indicated concentrations of D22 (*n* = 12). **(B)** Depicts normalized L_L_/L_1_ values of spontaneous [^3^H] outflow in presence of the indicated concentrations of D22 (*n* = 12). Significance of differences was evaluated by Kruskal-Wallis tests followed by Dunn’s multiple comparisons; ** and *** indicate *p* < 0.01 and *p* < 0.001 vs. solvent, respectively.

While 3 µM D22 inhibited MPP^+^ uptake by about 50%, the reduction of electrically evoked MPP^+^ efflux by this concentration was almost complete. To find out whether some lower concentrations of D22 might cause an enhancement of electrically evoked MPP^+^ release, 0.1–1 µM D22 were tested for such actions as well. The resulting concentration response relation showed that 1 µM D22 reduced action potential dependent release by about 50%, whereas lower concentrations failed to cause significant alterations. The respective concentration response relation revealed half maximal inhibition at 1.4 (1.0–1.9) µM (nH = 1.95; *R* ^2^ = 0.6; Figure 3A). Spontaneous outflow of MPP^+^, in contrast, displayed a concentration dependent increase in the presence of D22 (Figure 3B).

### 3.3 Effects of D22 on action potentials in SCG neurons

**FIGURE 4:**
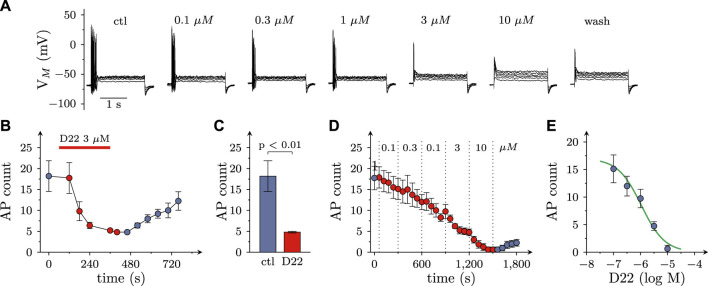
Effects of D22 on action potential firing in SCG neurons. Membrane potential of single SCG neurons was determined in current clamp using the perforated patch configuration. Action potential (AP) firing was elicited by injection of six depolarizing 2 s currents with amplitudes ranging from 50 pA to 300 pA in 50 pA increments once every 10 s. **(A)** Sample traces of current clamp recordings in one SCG neuron in presence of either solvent (control; ctl) or the indicated concentrations of D22 and after removal of D22 (wash). Traces were obtained at the end of 300 s exposure periods. **(B)** Time course of changes in numbers of action potentials (AP count) fired in response to current injections before, during, and after a 300 s application period of 3 µM D22 (*n* = 5). **(C)** Numbers of action potentials (AP count) fired in response to current injections just before and at the end of a 300 s application period of 3 µM D22 (*n* = 5, Mann-Whitney *U*-test). **(D)** Time course of changes in numbers of action potentials (AP count) fired in response to current injections before, during, and after 300 s application periods of the indicated D22 concentrations (*n* = 8). **(E)** Concentration response relation for the reduction in numbers of action potentials (AP count) determined as shown in **(D)**.

As uptake inhibitors such as cocaine (Boehm et al., 1995) as well as corticosterone and β-estradiol (see above) most commonly cause an augmentation of action potential dependent transmitter release from SCG cultures, the observation of an inhibitory effect of D22 was rather unexpected. To reveal whether this discrepancy might be related to effects other than uptake inhibition, SCG neuron excitability was investigated. To this end, action potentials were triggered by injections of 6 depolarizing current pulses with increasing amplitudes delivered once per minute in the absence and presence of D22, respectively. As to be seen in Figure 4A, numbers of action potentials fired in response to such current pulses were progressively reduced when escalating concentrations of D22 were applied; at 10 μM, no more action potentials were seen (Figures 4A, C). The resulting concentration response curve revealed half maximal inhibition at 1.1 (0.5–2.2) µM D22 (*R* ^2^ = 0.6; Figures 4D, E). To investigate the time course of this effect in more detail, 3 µM D22 was present for periods of 5 min; during these periods, numbers of action potentials per 6 current pulses were diminished increasingly to reach a plateau after 4 min; after removal of D22, this number slowly increased again towards baseline (Figure 4B).

### 3.4 Effects of D22 on voltage activated Ca^2+^ currents in SCG neurons

**FIGURE 5:**
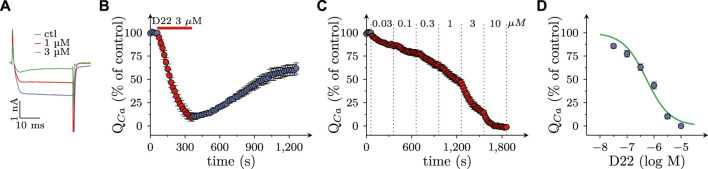
Effects of D22 on calcium currents in SCG neurons. Voltage activated calcium currents of single SCG neurons were determined in voltage clamp using the perforated patch configuration. From a holding potential of to −80 mV, neurons were depolarized to +10 mV for 30 ms once every 15 s. **(A)** Sample traces of voltage clamp recordings in one SCG neuron in presence of either solvent (control; ctl) or the indicated concentrations of D22. **(B)** Time course of percent changes in charge transfer via calcium currents (Q_Ca_) before, during, and after a 300 s application period of 3 µM D22 (*n* = 5). **(C)** Time course of percent changes in charge transfer via calcium currents (Q_Ca_) before and during 300 s application periods of the indicated D22 concentrations (*n* = 9). **(D)** Concentration response relation for the percentage reduction in charge transfer via calcium currents (Q_Ca_) determined as shown in **(D)**.

Not only the prevention of action potential propagation, but also the blockage of voltage activated Ca^2+^ channels can suffice to impede electrically evoked transmitter release from SCG neurons (Boehm, 1999). Currents through such channels were triggered by 30 ms depolarizations from −80 to +10 mV at a frequency of 4 per minute in perforated patch recordings (Figure 5A). In the presence of 3 µM D22, current amplitudes steadily declined and reached an apparent minimum after 5 min. Upon removal of D22 amplitudes slowly returned towards baseline, but recovery remained incomplete even after 15 min of washout (Figure 5B). When increasing concentrations of D22 were applied, current amplitudes were progressively reduced, and at 10 µM no more current was visible (Figures 5A, C). The resulting concentration response curve showed half maximal inhibition of Ca^2+^ currents at 0.5 (0.4–0.6) µM D22 (*R* ^2^ = 0.9; Figure 5D).

### 3.5 Effects of D22 on K^+^ evoked release of MPP^+^ from SCG neurons: comparison with corticosterone

**FIGURE 6:**
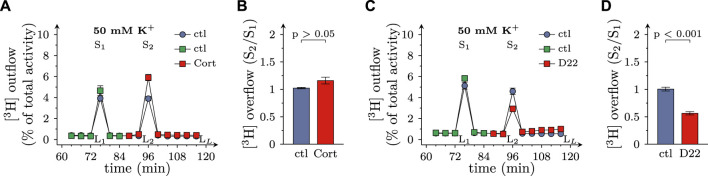
Effects of D22 on K^+^ evoked release of [^3^H]MPP^+^ from SCG neurons: comparison with corticosterone. SCG cultures were labeled with [^3^H]MPP^+^ and superfused; subsequent to a 60 min washout period, 4 min fractions of superfusate were collected. 50 mM KCl (NaCl was reduced accordingly) was present in minutes 76 (S_1_) and 96 (S_2_), respectively. Corticosterone (Cort; 100 µM), D22 (3 µM), or solvent (control; ctl) were present from minute 84 onward as indicated. **(A,C)** show the time-course of [^3^H] outflow as determined in representative experiments (*n* = 3 each). **(B,D)** depict S_2_/S_1_ values of K^+^ evoked [^3^H] overflow in presence of solvent or uptake inhibitors as indicated (*n* = 9 each, Mann-Whitney *U*-test). L_L_/L_1_ values of spontaneous [^3^H] outflow in these experiments are contained in Figures 2C, I, respectively.

When transmitter release from SCG neurons is triggered by depolarizing K^+^ concentrations, voltage activated Ca^2+^ channels, but not action potential propagation, are involved in stimulus secretion coupling (Boehm, 1999). In the present experiments, K^+^ evoked release of MPP^+^ tended to get enhanced by 100 µM corticosterone (even though this effect failed to reach statistical significance), but was reduced by 3 µM D22 (Figures 6A–D). Thus, there is a similar discrepancy between the effects of these two blockers of OCTs as observed with electrically evoked release of MPP^+^ before.

## 4 Discussion

In light of the increasing perception of OCTs and PMAT as key players in monoaminergic pathways of the nervous system, blockers of these transporters have gained growing attention by the respective research community (Bönisch, 2021; Koepsell, 2021). The only transport inhibitor with micromolar affinities for OCTs as well as PMAT is D22 (Bönisch, 2021; Koepsell, 2021), and this agent has been employed in numerous *in vitro* (Hosford et al., 2014; Lloyd et al., 2019; Lee et al., 2021) and *in vivo* (Horton et al., 2013; Hosford et al., 2014; Marcinkiewcz and Devine, 2015; Bowman et al., 2020) experiments in order to substantiate biological roles of these transporters. Here, D22 has been used to investigate functions of OCTs in primary cultures of SCG, and the results reveal effects of micromolar concentrations of this drug that are not related to transport inhibition.

### 4.1 Reuptake of MPP^+^ into SCG neurons via OCT3

Incorporation of MPP^+^ into primary cultures of rat SCG was reduced by approximately 70% by blockers of monoamine transporters (cocaine and desipramine) and by about 30% by blockers of OCTs (corticosterone and β-estradiol). Over 95% of this MPP^+^ uptake occurred in neurons with negligible contribution of other cells also present in primary SCG cultures. Blockage of either monoamine transporters or OCTs appeared to complement one another to likely result in full inhibition of neuronal MPP^+^ uptake when combined. In SCG cultures, expression of the noradrenaline and serotonin, but not the dopamine, transporter (Nishimura et al., 1999) and of OCT3, but not OCT1 or -2 (Kristufek et al., 2002) has been detected at the mRNA level. In SCG neurons in culture, NET is found almost exclusively at axons and boutons but hardly at neuronal somata (Matthies et al., 2009), and the former ones are the sites where vesicle exocytosis occurs (Boehm, 1999). The precise localization of OCT3 in these neurons has not been investigated so far.

In previous investigations, action potential dependent transmitter release has been found to be augmented by cocaine (Boehm et al., 1995). The present experiments proved that action potential dependent release of previously incorporated [^3^H]MPP^+^ was enhanced by two blockers of OCT3, corticosterone and β-estradiol. These two agents also elevated spontaneous outflow of radiotracer, an effect that has not been investigated in sufficient detail for cocaine (Boehm et al., 1995). Despite the fact that [^3^H]MPP^+^ has been used in the present experiments instead of [^3^H]noradrenaline, these results suggest that neurotransmitter reuptake after release from axonal boutons of SCG neurons may involve both, NET and OCT3. Previously, blockage of both, monoamine transporters by cocaine and OCT3 by corticosterone, has been shown to inhibit catecholamine reuptake after action potential dependent release from presynaptic terminals in the basolateral amygdala of the rat (Holleran et al., 2020).

### 4.2 Inhibition of MPP^+^ uptake into SCG neurons by D22

D22 reduced uptake of [^3^H]MPP^+^ in a concentration dependent manner with half maximal inhibition at 3 µM and approximately 80% inhibition at 10 µM. This contrasts with the just 30% uptake inhibition achieved by 100 µM corticosterone and 100 µM β-estradiol, respectively. The steroids display affinities at OCT3 in the low to sub-micromolar range, whereas that of D22 is approximately 0.1 µM (Bönisch, 2021; Koepsell, 2021). Hence, all the concentrations indicated above should yield complete inhibition of uptake via OCT3. Therefore, the virtually complete uptake inhibition by D22, as opposed to the limited actions of steroids, most likely involves transporters other than OCT3. In this context, one should bear in mind that micromolar concentrations of D22 have been reported to interfere with radiotracer uptake via all three monoamine transporters, with half maximal reduction of uptake via the mouse NET at 35 µM (Fraser-Spears et al., 2019). In addition, D22 is known to block PMAT with affinities of about 1 µM (Bönisch, 2021; Koepsell, 2021). Thus, the 80% reduction of uptake by 10 µM D22 must be assumed to involve an inhibition of NET and, in addition, might indicate a role of PMAT. However, PMAT expression or function has not been reported for SCG neurons.

### 4.3 Inhibition of depolarization dependent release of MPP^+^ from SCG neurons by D22

Spontaneous release of previously incorporated [^3^H]MPP^+^ was enhanced in the presence of D22 as it was in the presence of corticosterone and β-estradiol. In this regard, the most obvious explanation would be the underlying prevention of MPP^+^ reuptake by blockage of OCT3 as all three OCT3 inhibitors exerted analogous actions. As anticipated, depolarization dependent [^3^H]MPP^+^ overflow, in parallel to spontaneous radiotracer outflow, was enhanced by corticosterone and β-estradiol, respectively. Unexpectedly, depolarization dependent release of [^3^H]MPP^+^ was reduced by D22. The inhibition of electrically evoked release was half maximal at about 1 µM D22. Hence, inhibition of uptake and of release of MPP^+^ were observed at the same range of concentrations.

Previously, identical micromolar concentrations of D22 have been found to reduce release of [^3^H]MPP^+^ from SCG neurons when triggered by amphetamine, and this inhibitory action was concluded to be due to a block of OCT3 (Mayer et al., 2018). Nevertheless, it appears counterintuitive to assume that a diminution of depolarization evoked release, which relies on Ca^2+^ dependent vesicle exocytosis (see below), would be restricted by blockage of transport proteins, be it monoamine transporters, OCTs, or PMAT. Therefore, in order to explain these inhibitory effects of D22 alternative mechanisms of action had to be uncovered.

### 4.4 Block of action potentials and voltage activated Ca^2+^ channels by D22

In perforated patch recordings on single SCG neurons, D22 decreased the numbers of action potentials elicited by injections of depolarizing currents, on the one hand, and diminished charge transfer via currents through voltage activated Ca^2+^ channels, on the other. Both effects displayed almost identical concentration response curves with half maximal inhibition at 1 µM (action potentials) and 3 µM (Ca^2+^ currents), respectively, and complete abolition at 10 µM. Thus, these two effects occurred within the same range of concentrations as the reduction of electrically evoked release of MPP^+^. For comparison, D22 blocks OCT3 and PMAT with affinities of about 0.1–1 µM (Bönisch, 2021; Koepsell, 2021).

As methods to evoke depolarization dependent release of previously incorporated [^3^H]MPP^+^, electrical field stimulation and 50 mM K^+^ have been used, respectively. While the former triggers action potential propagation along the axons, the latter leads to tonic depolarization of presynaptic boutons. Accordingly, only electrically, but not K^+^-evoked radiotracer release is sensitive towards Na^+^ channel blockage by, for instance, tetrodotoxin. With both types of stimulation, however, blockers of voltage activated Ca^2+^ channels, such as Cd^2+^, prevent radiotracer overflow (Boehm, 1999). These facts allude to vesicle exocytosis, and not reverse transport, as underlying process and to transmembrane Ca^2+^ influx as final ionic mechanism that leads to activation of synaptotagmins and clompexins to permit Ca^2+^-dependent fusion between vesicle and presynaptic plasma membranes (Südhof, 2013). Even though Ca^2+^ currents were reduced by D22 at somewhat higher concentrations than action potential firing, voltage-activated Ca^2+^ rather than Na^+^ channels can be viewed as key targets for D22 to interfere with transmitter release, as the former provide a direct link to vesicle exocytosis.

Previously, D22 at 25 µM has been found to reduce spontaneous firing of action potentials in neurons located within substantia nigra pars compacta of mouse horizontal midbrain slices. In those experiments, evidence for inhibitory effects of such high D22 concentrations on G protein-gated inwardly rectifying K^+^ channels as well as HCN channels has been obtained as well (Lloyd et al., 2019). In contrast, evidence for inhibitory actions of D22 on voltage-activated Ca^2+^ channels has not been presented so far. Nevertheless, in pinealocytes in cell culture D22 was found to reduce release of serotonin, but the authors argued that the mechanism mediating release was not exocytosis, but rather outward transport via PMAT (Lee et al., 2021). Therefore, the present report is the first to reveal that D22 may reduce exocytotic transmitter release through an inhibition of voltage-activated Ca^2+^ channels.

In this context, the molecular identity of Ca^2+^ channels that mediate depolarization evoked currents as well as transmitter release in rat SCG neuron should be discussed briefly. A major proportion of 70%–75% (Kubista et al., 2009) is mediated by Cav2.2 channels, and the remainder involves Cav2.1 channels and members of the Cav1 family (Boehm and Kubista, 2002). As Ca^2+^ currents were abolished completely in the presence of 10 µM D22, this action does not appear to be specific for certain channel subtypes. At odds with the present findings is the observation that the presence of 10 µM D22 did not reduce amplitudes of action potential evoked excitatory postsynaptic currents in neurons of the nucleus tractus solitarii within rat brainstem slices (Hosford et al., 2014). The underlying release of glutamate from nerve terminals of tractus solitarius relies to a large extent on the gating of Cav2.2.channels (Mendelowitz et al., 1995). The reason for this discrepancy between those and SCG neuron axon terminals as investigated here is not obvious, but could be related to the fact that concentrations of drugs that reach their targets in brain slices may be lower than those applied to the surface.

### 4.5 Transporter-independent effects of D22

Inhibition of transmitter reuptake after exocytotic release from postganglionic sympathetic axonal boutons whether by NET or OCT3 blockage can be expected to lead to indirect sympathomimetic effects. Accordingly, cocaine is known to raise blood pressure in rats (Pitts et al., 1987). D22, in contrast, led to an unexpected drop in blood pressure, and this was due to antagonistic actions at α_1_ adrenergic receptors (Russ et al., 1996). In this context, one should not forget that blockers of Cav1 channels lead to a reduction of blood pressure as well (Daghbouche-Rubio et al., 2022). Moreover, cocaine’s psychostimulant activity causes hyperlocomotion (Uhl et al., 2002), whereas D22 is known to reduce locomotor activity by a yet unidentified mechanism (Krause-Heuer et al., 2017). In analogy to these previous reports of results obtained with D22 that were not related to inhibition of monoamine reuptake, the present data indicate that this blocker of OCT3 and PMAT can reduce depolarization dependent release of catecholamines instead of causing an enhancement as expected for a transport inhibitor. This inhibitory effect can be correlated with an inhibition of voltage activated Ca^2+^ channels and an interruption of action potential propagation most likely by a block of voltage activated Na^+^ channels. Together with the suppression of currents through inward rectifier K^+^ and HCN channels as shown before (Lloyd et al., 2019), this firmly establishes various types of ion channels as potential targets for D22. Conceivably, the suppression of ionic currents can lead to changes in neuronal activity to varying degrees across different brain areas. This, in turn, may impact animal behavior and thereby contribute to *in vivo* actions of D22 such as antidepressant activity as observed repeatedly (Horton et al., 2013; Marcinkiewcz and Devine, 2015; Bowman et al., 2020). Therefore, results obtained with D22 need to be interpreted cautiously as this cyanine derivative can produce several effects in the nervous system with no relation to neurotransmitter transport whatsoever.

## Data Availability

The raw data supporting the conclusion of this article will be made available by the authors, without undue reservation.
